# Editorial: Heparan sulfate-binding proteins in health and disease

**DOI:** 10.3389/fmolb.2024.1386623

**Published:** 2024-03-20

**Authors:** Lauren A. Gandy, Fuming Zhang, Ding Xu, Lars C. Pedersen, Kay Grobe, Chunyu Wang

**Affiliations:** ^1^ Shirley Ann Jackson, Ph.D. Center for Biotechnology and Interdisciplinary Studies, Rensselaer Polytechnic Institute, Troy, NY, United States; ^2^ Glycobiology, Cell Growth and Tissue Repair Research Unit (Gly-CRRET), Université Paris-est Créteil, Créteil, France; ^3^ Department of Oral Biology, School of Dental Medicine, University of Buffalo, The State University of New York, Buffalo, NY, United States; ^4^ Genome Integrity and Structural Biology Laboratory, National Institute of Environmental Health Sciences, National Institutes of Health, Durham, NC, United States; ^5^ Institute of Physiological Chemistry and Pathobiochemistry, University of Münster, Münster, Germany; ^6^ Department of Biology, Troy, NY, United States; ^7^ Department of Chemistry and Chemical Biology, Rensselaer Polytechnic Institute, Troy, NY, United States

**Keywords:** heparan sulfate (HS), heparin, heparin-binding proteins, glycosaminoglycan (GAG), fucoidan (FPS), tauopathies, hedgehog autoprocessing, sepsis

This Research Topic is dedicated to a distinguished figure in the heparan sulfate (HS) field, Professor Robert J. Linhardt, whose contributions have left an indelible mark. Recognized globally for his pioneering research in HS and heparin, Dr. Linhardt’s expertise has established him as a foremost authority in glycan analysis and sequencing with mass spectrometry, SPR, NMR, and nanopore technology. His notable achievements include being the first to sequence the glycosaminoglycan chain of a proteoglycan (Ly et al., 2011) and contributions to the synthesis of a glycosaminoglycan through metabolic engineering in *E. coli* (Badri et al., 2021). His work has been pivotal in the development of heparin-related drugs, including tinzaparin, aradaparin, enoxaparins, and low molecular weight heparins. During the 2007–8 heparin contamination crisis, he spearheaded efforts to quickly identify the molecular culprit as oversulfated chondroitin sulfate (Liu et al., 2009). For this crucial contribution, he was named as one of the Scientific American 10: Guiding Science for Humanity in 2009 (https://www.scientificamerican.com/article/scientific-american-10/). Dr. Linhardt’s prolific output includes over 1,500 scientific publications with an H-factor of 138, and he holds more than 50 patents. Despite his recent retirement, he continues to publish valuable contributions to the field. His dedication to education is equally impressive, having mentored 81 Ph.D. and 15 master’s students throughout his career, shaping the next-generation of scientists. He has been a mentor, colleague, and friend to many in the glycan field.

We are honored to carry on Dr. Linhardt’s legacy by presenting six original articles and two reviews on HS-binding proteins (HSBP). HSBPs play essential roles in many physiological processes such as signal transduction, blood coagulation and immune response (Möckl, 2020). They are also critically involved in many diseases, including the cellular entry of pathogens, prion-like spread of amyloids, sepsis and nephritis.

Li et al. begins our Research Topic with an insightful review of structural mechanisms of HS/protein interaction, and how HS/protein interactions can be targeted by HS-based oligosaccharides and monoclonal antibodies, making an excellent case for using mAb to disrupt specific HSBP interactions; while Faris et al. demonstrated the utility of a novel AlphaScreen assay for discovering inhibitors of protein-HS complexes, by targeting tau-HS interaction in Alzheimer’s disease. Liao et al. presents an in-depth review of the involvement of HSBP in sepsis, highlighting the role of HMGB1 and the therapeutic potential of chemoenzymatically synthesized HS oligosaccharides in sepsis. Buijsers et al. demonstrated the protective effects of HS and fucoidan in kidney disease.

These are followed by an SAR study of marine sulfated glycan in antithrombin and PF4 binding for coagulation in Zhang et al. Gandy et al. delved into why herpes virus requires the rare 3-*O*-sulfation modification for HS-mediated viral entry, discovering that the presence of this sulfation group shortens the HS length requirement for recognition by herpes glycoprotein D. Finally, Manikowski et al. demonstrated that HS plays an important role in the range of Hh ligand signaling in *Drosophila* wing development.

As shown in this excellent Research Topic collection, HSBPs are involved in a large number of biological and pathological processes. The field of HSBP is expanding rapidly, as our knowledge of HS and HSBPs in health and disease grows. Since the characterization of antithrombin and HS in the early 1980s (Petitou et al., 2003; Shriver et al., 2012), investigations of HS-HSBP interactions have provided crucial insights into some of the most complex diseases, such as Alzheimer’s disease (Holmes et al., 2013; Zhao et al., 2020; Mah et al., 2021), HPV (Johnson et al., 2009; Shafti-Keramat et al., 2003), and SARS-COV-2 (Clausen et al., 2020; Yue et al., 2021; Kearns et al., 2022). Further characterization of the involvement of HS/HSBP in health and disease will provide novel mechanistic insights and hopefully therapeutic opportunities to improve human health.

## Author note’s

This article is dedicated to Dr. Robert J. Linhardt.
